# Targeting of HSP70/HSF1 Axis Abrogates In Vitro Ibrutinib-Resistance in Chronic Lymphocytic Leukemia

**DOI:** 10.3390/cancers13215453

**Published:** 2021-10-29

**Authors:** Federica Frezzato, Andrea Visentin, Filippo Severin, Serena Pizzo, Edoardo Ruggeri, Nayla Mouawad, Leonardo Martinello, Elisa Pagnin, Valentina Trimarco, Alessia Tonini, Samuela Carraro, Stefano Pravato, Silvia Imbergamo, Sabrina Manni, Francesco Piazza, Anna Maria Brunati, Monica Facco, Livio Trentin

**Affiliations:** 1 Department of Medicine, Hematology and Clinical Immunology Branch, Padua University School of Medicine, 35128 Padua, Italy; federica.frezzato@unipd.it (F.F.); andrea.visentin@aopd.veneto.it (A.V.); filippo.severin@unipd.it (F.S.); serena.pizzo.1@phd.unipd.it (S.P.); edoardo.ruggeri@phd.unipd.it (E.R.); nayla.mouawad@unipd.it (N.M.); leonardo.martinello@studenti.unipd.it (L.M.); elisa.pagnin@unipd.it (E.P.); valentina.trimarco@unipd.it (V.T.); alessia.tonini@aopd.veneto.it (A.T.); samuela.carraro@unipd.it (S.C.); stafano.pravato@aopd.veneto.it (S.P.); silvia.imbergamo@aopd.veneto.it (S.I.); sabrina.manni@unipd.it (S.M.); francesco.piazza@unipd.it (F.P.); monica.facco@unipd.it (M.F.); 2 Laboratory of Myeloma and Lymphoma Pathobiology, Veneto Institute of Molecular Medicine (VIMM), 35129 Padua, Italy; 3 Department of Molecular Medicine, Padua University, 35121 Padua, Italy; annamaria.brunati@unipd.it

**Keywords:** chronic lymphocytic leukemia, Ibrutinib, HSP70, HSF1, drug-resistance, phenols

## Abstract

**Simple Summary:**

The use of ibrutinib has changed the management and clinical history of patients with multiple-treated chronic lymphocytic leukemia (CLL). Nevertheless, an increasing number of patients develop resistance to treatment, with mechanisms still to be fully clarified. Since HSP70 plays a pivotal role in mediating the survival and the progression of CLL, we herein addressed the role of HSP70 and its regulator HSF1 in the development of ibrutinib-mediated resistance. We found an increase in both proteins when the treatment was failing, and thus the disease was progressing. This suggests the involvement of HSP70 in mechanisms of drug resistance. Moreover, we demonstrated that the use, at different levels, of HSP70/HSF1 axis inhibitors could represent a novel rational therapeutic approach to overcome ibrutinib resistance in those patients who relapsed after this type of treatment.

**Abstract:**

The Btk inhibitor ibrutinib has significantly changed the management of chronic lymphocytic leukemia (CLL) patients. Despite its clinical efficacy, relapses occur, and outcomes after ibrutinib failure are poor. Although BTK and PLCγ2 mutations have been found to be associated with ibrutinib resistance in a fair percentage of CLL patients, no information on resistance mechanisms is available in patients lacking these mutations. The heat shock protein of 70 kDa (HSP70) and its transcription factor heat shock factor 1 (HSF1) play a role in mediating the survival and progression of CLL, as well as taking part in drug resistance in various cancers. We demonstrated that resveratrol and related phenols were able to induce apoptosis in vitro in leukemic cells from CLL untreated patients by acting on the HSP70/HSF1 axis. The same was achieved in cells recovered from 13 CLL patients failing in vivo ibrutinib treatment. HSP70 and HSF1 levels decreased following in vitro treatment, correlating to apoptosis induction. We suggest an involvement of HSP70/HSF1 axis in controlling resistance to ibrutinib in CLL cells, since their inhibition is effective in inducing in vitro apoptosis in cells from ibrutinib refractory patients. The targeting of HSP70/HSF1 axis could represent a novel rational therapeutic strategy for CLL, also for relapsing patients.

## 1. Introduction

Chronic lymphocytic leukemia (CLL) is a neoplastic disorder of the elders, characterized by the accumulation of clonal B lymphocytes, due to uncontrolled growth and resistance to apoptosis. CLL is a genetically complex disease that affects a heterogeneous patient population. Therapeutic armamentarium of CLL changed recently from the introduction of novel active agents [1]. In particular, the introduction of Bruton’s tyrosine kinase (Btk) inhibitor ibrutinib has significantly changed the management of patients with CLL, achieving extremely high efficacy even in high-risk and chemo-refractory patients.

Ibrutinib is thought to also be a promising target drug for diffuse large B-cell lymphoma (DLBCL) [2,3], and is now FDA-approved for relapsed/refractory mantle cell lymphoma (MCL), Waldendström macroglobulinemia (WM), marginal zone lymphoma (MZL), and, of course, as both a first- and second-line therapy for CLL [4,5,6].

Although the encouraging efficacy of ibrutinib in the majority of CLL patients demonstrates a durable response, relapses do occur, and outcomes after ibrutinib failure are dismal due to the lack of effective agents [7]. Even though patients who experience ibrutinib relapse can be treated with other compounds (i.e., venetoclax, acalabrutinib, zanubrutinib), the need for new therapies still remains. Little is known about the protein adaptation that could mediate or favor the development of resistance to ibrutinib. Mutations affecting Btk residue C481, which is bound covalently by ibrutinib, or phospholipase Cγ2 (PLCγ2), an immediate downstream target of Btk, or activation of non-classical NF-κB signalling were identified in patients who progressed or were intrinsic resistant to ibrutinib [8]. However, these mutations are not univocal, indicating that additional mechanisms remain to be clarified.

We previously demonstrated that chaperone adaptor heat shock protein of 70 kDa (HSP70) and its transcription factor heat shock factor 1 (HSF1) play a pivotal role in mediating the survival and the progression of CLL [9,10]. Moreover, in several cancers, including hematological neoplasias, both proteins have been indicated to be involved in drug-resistance [11,12,13]. Our group also demonstrated a correlation between HSP70 levels and the response to chemo-immunotherapy (i.e., fludarabine and cyclophosphamide, plus rituximab or bendamustine, plus rituximab) [9]. In fact, HSP70 decreased only in those patients responding to therapy, but not in non-responder patients. Of note, in patients who were responding to ibrutinib-containing regimen, HSP70 and HSF1 were consensually reduced after therapy [10].

The overexpression of HSP70 and HSF1 in tumors, including CLL, is not surprising, since cancer cells, characterized by higher metabolic needs and aberrantly activated signaling pathways, show a higher demand for chaperone cytoprotective functions. In highly tumorigenic cells, HSF1 transcriptional activity regulates cancer-specific genes, which are different from genes activated during physiological processes [14]. HSF1 activation, via phosphorylation of Ser326, precedes malignancy, allowing cells to withstand a wide range of proteotoxic insults, even during the early stages of carcinogenesis. In stressed cells, HSF1 is hyperphosphorylated, and this might be essential for the regulation of gene transcription [15,16]. When cells are subjected to pro-malignant signaling, HSF1 is de-phosphorylated on Ser303 and phosphorylated on Ser326. We found that CLL patients showing high levels of HSP70 also expressed high Akt-Ser473, which activates HSF1. In contrast, patients with low HSP70 displayed high activation of MEK1/2 and ERK1/2, known to negatively regulate HSF1 [10]. In CLL, this evidence shows that HSP70 expression is regulated by the modulation of HSF1 activity through the activation of RAS-regulated pathways (as previously observed in CML [17] and MM [18]), and suggests the HSP70/HSF1 interplay as an interesting target for anti-leukemic therapies. HSP70 and HSF1 inhibition, in fact, has been proven to be effective in inducing a dose-dependent in vitro apoptosis of CLL cells [9,10]. Moreover, supporting the implication of HSP70/HSF1 axis in Ibrutinib-resistance in CLL, PI3K/Akt pathway has been found to be hyper-activated in those patients [19,20].

In recent years, the potential of the use of small natural molecules to prevent and remediate negative effects of both pathological conditions and cancers was deeply studied [21,22]. Of note, flavonoids or non-flavonoids polyphenols have been demonstrated to display health benefits in terms of anti-oxidant, -inflammatory, -cancer, -diabetic, -osteoporosis, -obesity, and -hypertensive activities, as well as being hepato- and neuro-protective, [23] and even as specific inhibitors (i.e., quercetin) of SARS-CoV2, the virus responsible for COVID-19 (coronavirus disease 2019) [24]. In particular, the hypothesized beneficial properties of resveratrol have led to an increase in its consumption in food supplements; moreover, over the past 20 years, resveratrol has been the most studied polyphenol with anti-leukemia activity [25]. Resveratrol, as well as correlated molecules (i.e., pterostilbene, triacetyl resveratrol and honokiol), is a stilbenoid polyphenol acting with an anti-kinase potential by contemporary inhibiting AKT and activating ERK [17], thus potentially overall inhibiting HSF1 and, in turn, HSP70.

In this context, we correlated HSP70 and HSF1 expression levels and response to Ibrutinib-containing regimen in CLL patients, demonstrating that these two molecules were overexpressed before or during progression in patients failing ibrutinib. Moreover, we assessed the effect of the inhibition of HSP70/HSF1 axis by the targeting of RAS-signalling pathway, demonstrating its efficacy in CLL cells.

## 2. Results

### 2.1. Resveratrol Induces Apoptosis in CLL Cells by Targeting HSP70/HS1 Axis

**Figure 1:**
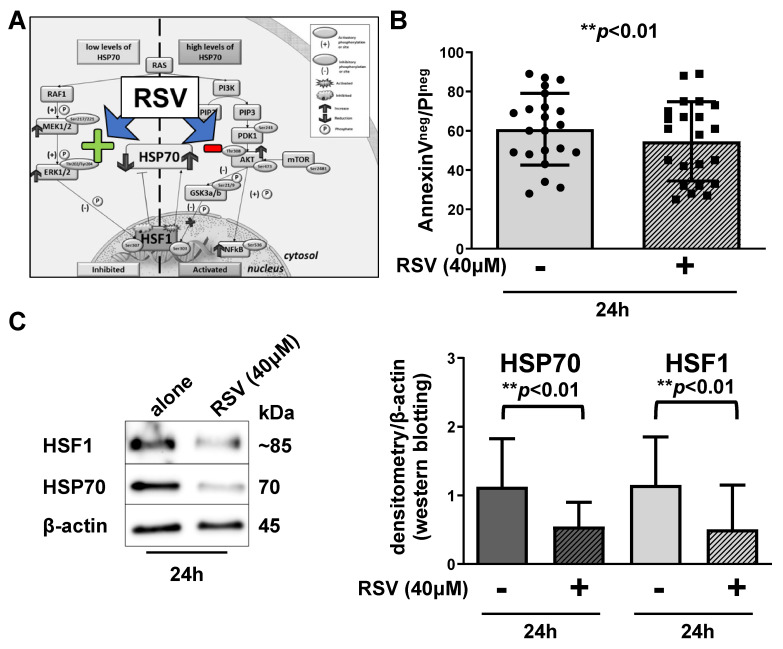
Resveratrol induces apoptosis in CLL leukemic B cells. (**A**). The model explains HSP70 regulation by HSF1 via RAS-dependent pathways in CLL (adapted from 10) and RSV targets. In HSP70^high^ patients, we found an increased phosphorylation of AKT-Ser473 (↑) that is reflected in a high amount of GSK3a/b phosphorylated at inhibitory Ser21/9. In this way, GSK3a/b is no more able to inhibit HSF1 that, in turn, up-regulates HSP70 (↑). In HSP70^low^ patients, we found an up-regulation of the RAS-RAF-MEK1/2-ERK1/2 pathway; activated ERK at Thr202/Tyr204 (↑) can phosphorylate HSF1 at inhibitory Ser307, thus preventing HSP70 transcription (↓). RSV acts by inhibiting AKT (red minus) and by activating Erk (green plus). (**B**). Cells were cultured alone or in the presence of 40 µM RSV, and cell apoptosis was analyzed by annexin V/PI flow cytometric test. Histograms report the percentage of Annexin V^neg^/PI^neg^ cells after 24 h treatment. Data are reported as mean ± SD (** *p* < 0.01, paired Student’s *t* test; *n* = 22). (**C**)**.** Lysates obtained from CLL B cells cultured alone or with 40 µM RSV were analyzed by immunostaining with anti-HSP70, anti-HSF1 and anti-β-Actin as loading control. Western blotting is representative of all the cases analyzed (*n* = 11). Histograms represents the densitometry of HSP70/actin and HSF1/actin ratios of the 11 CLL samples analyzed (** *p* < 0.01, paired Student’s *t* test between the treated vs. the untreated condition). RSV: resveratrol. PI: propidium iodide.

Since most HSF1-phosphorylating molecules belong to two RAS-signaling pathways (PI3K/AKT/mTOR and RAF/MEK/ERK), taking advantage of a previous RPPA analysis [9], we correlated HSP70 expression to different proteins related to these pathways. By cluster analysis, we divided our patients into HSP70^high^ and HSP70^low^ considering as cut-off value the median of HSP70 expression levels calculated by RPPA. We demonstrated that the tested proteins are regulated in a different way between HSP70^high^ and HSP70^low^ patients, and we developed a model to explain this difference [10]. Patients with high expression of HSP70 have high AKT-Ser473, which inhibits GSK3a/b. In its inhibited form, GSK3a/b is no more able to inhibit HSF1. On the other hand, HSP70^low^ patients present high MEK1/2-Ser217/221 and ERK-Thr202/Tyr204, known to negatively regulate HSF1 [26]. To simultaneously affect the two RAS-mediated pathways, we used resveratrol (RSV), a natural polyphenol with anti-tumorigenic and anti-oxidant properties [27], able to inhibit AKT and activate ERK (Figure 1A). After 24h treatment with 40 µM RSV, we observed a reduction in cell viability (54 ± 20% of living cells) with respect to untreated cells (61 ± 18%; *p* < 0.01, paired Student’s *t* test—Figure 1B). The analysis of HSF1 and HSP70 expression levels showed that HSF1 decreased from 1.15 ± 0.69 to 0.50 ± 0.64 and HSP70 from 1.13 ± 0.69 to 0.54 ± 0.35, in CLL cells cultured alone and with 40 µM RSV, respectively (*p* < 0.01, paired Student’s *t* test—Figure 1C).

### 2.2. Resveratrol-Associated Molecules Induce Apoptosis in CLL Cells by Targeting HSP70/HS1 Axis

**Figure 2:**
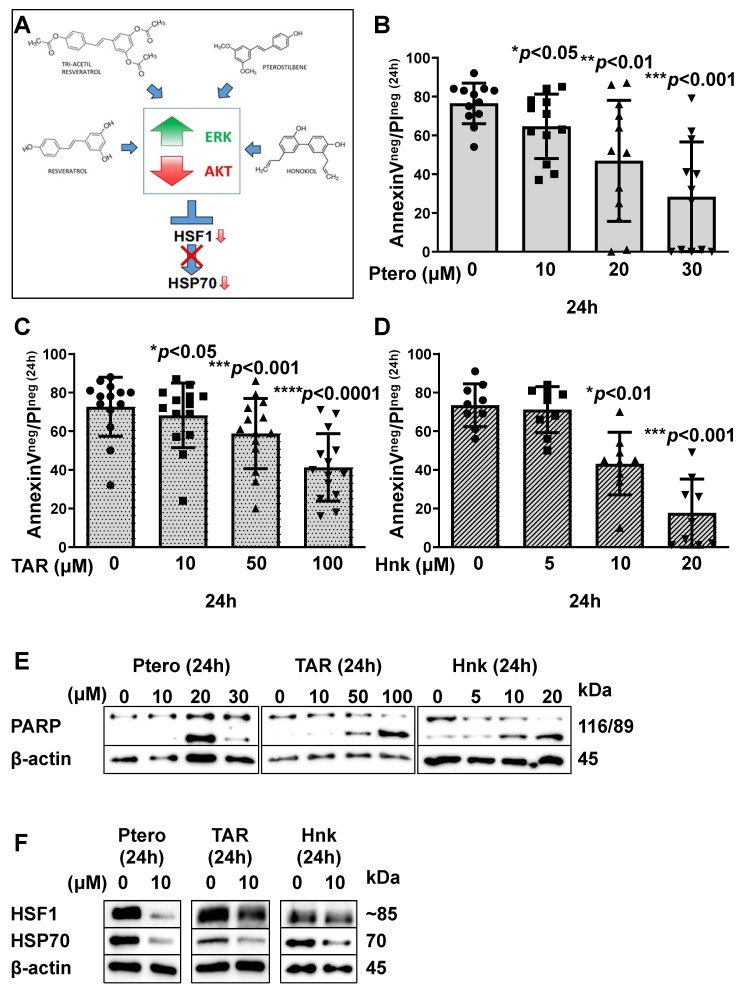
Pterostilbene, triacetyl resveratrol and honokiol induce apoptosis in CLL leukemic B cells. (**A**). Chemical structures of resveratrol, pterostilbene, triacetyl resveratrol and honokiol, and a schematic way of their action on ERK and AKT activation, and finally on HSP70/HSF1 axis. (**B**). Cells were cultured alone or in the presence of 10, 20 or 30 µM Ptero and cell apoptosis were analyzed by annexin V-PI flow cytometric test. Histograms report the percentage of annexin V^neg^/PI^neg^ cells after 24 h treatment. Data are reported as mean ± SD (* *p* < 0.05, ** *p* < 0.01 and *** *p* < 0.001 vs. the untreated condition, paired Student’s *t* test; *n* = 12). (**C**). Cells were cultured alone or in the presence of 10, 50 or 100 µM TAR and cell apoptosis were analyzed by annexin V/PI flow cytometric test. Histograms report the percentage of annexin V^neg^/PI^neg^ cells after 24 h treatment. Data are reported as mean ± SD (* *p* < 0.05, *** *p* < 0.001 and **** *p* < 0.0001 vs. the untreated condition, paired Student’s *t* test; *n* = 14). (**D**). Cells were cultured alone or in the presence of 5, 10 or 20 µM Hnk, and cell apoptosis was analyzed by annexin V-PI flow cytometric test. Histograms report the percentage of Annexin V^neg^/PI^neg^ cells after 24 h treatment. Data are reported as mean ± SD (*** *p* < 0.001 and **** *p* < 0.0001 vs. the untreated condition, paired Student’s *t* test; *n* = 9). (**E**). Lysates obtained from CLL B cells cultured for 24 h alone or with drug concentrations listed in previous points B., C., and D., were analyzed by immunostaining with anti-PARP, for apoptosis detection, and anti-β-Actin as loading control. Western blotting is representative of all the cases analyzed. (**F**). Lysates obtained from CLL B cells cultured for 24 h alone or with 10 µM Ptero, 10 µM TAR and 10 µM Hnk were analyzed by immunostaining with anti-HSP70, anti-HSF1 and anti-β-Actin as loading control. Western blotting is representative of all the cases analyzed. Ptero: pterostilbene; TAR: triacetyl resveratrol; Hnk: honokiol.

Despite its in vitro efficacy in CLL, RSV has low bioavailability and poor potency. For this reason, we performed the same experiments with other inhibitors presenting a structure and action similar to resveratrol named pterostilbene (Ptero; a stilbenoid found in almonds, grape leaves and vines and blueberries); triacetyl resveratrol (TAR; displaying superior bioavailability to the parent compound, resveratrol); honokiol (Hnk; a poly-phenolic compound isolated from the bark, seed cones, and leaves of trees belonging to the genus Magnolia). Considering their structures and actions, like those of RSV, these compounds are supposed to have a final effect on HSP70 and HSF1 (Figure 2A). Freshly isolated cells from untreated CLL patients have been cultured for 24 h with Ptero 10 µM, 20 µM and 30 µM. The compound was effective starting from 10 µM (63.50% ± 14.47% of viable cells) with respect to untreated cells (74.63% ± 11.77%; *p* < 0.05; paired Student’s *t* test). As expected, a greater degree of apoptosis was observed at 20 µM (48.50% ± 21.59%; *p* < 0.05; paired Student’s *t* test) and 30 µM (24.88% ± 22.03%; *p* < 0.01; paired Student’s *t* test—Figure 2B). As for TAR, cells from 14 untreated patients were cultured for 24 h with different concentrations of this compound, which showed to be effective at 10 µM (68.14% ± 16.72% of viable cells) with respect to untreated cells (72.64% ± 15.30%; *p* < 0.05; paired Student’s *t* test). Cell viability further decreased at 50 µM (58.79% ± 18.18%; *p* < 0.001, paired Student’s *t* test) and 100 µM (41.29% ± 17.45%; *p* < 0.0001, paired Student’s *t* test—Figure 2C). Finally, freshly isolated cells from 9 CLL untreated patients were cultured for 24 h with Hnk 5 µM, 10 µM, and 20 µM. Decrease in cell viability was significant at 10 µM (45.70% ± 17.13%; *p* < 0.01, paired Student’s *t* test) and 20 µM (23.80% ± 25.54%; *p* < 0.001 paired Student’s *t* test) with respect to the untreated condition (73.40% ± 10.45%—Figure 2D). Apoptosis induction has been assessed by Western blot analysis of cleaved PARP (Figure 2E). For all the samples examined, a reduction of both HSP70 and HSF1 was demonstrated for all the treated conditions (Figure 2F). When we subjected B cells obtained from healthy controls to the same treatments (i.e., RSV, Ptero, TAR and Hnk), we did not observe any difference in cell viability after 24 h, as assessed by Wilcoxon test (*p* = ns, Figure S1).

### 2.3. HSP70 and HSF1 Expression Levels Are Increased in Patients Who Failed Ibrutinib Treatment

**Figure 3:**
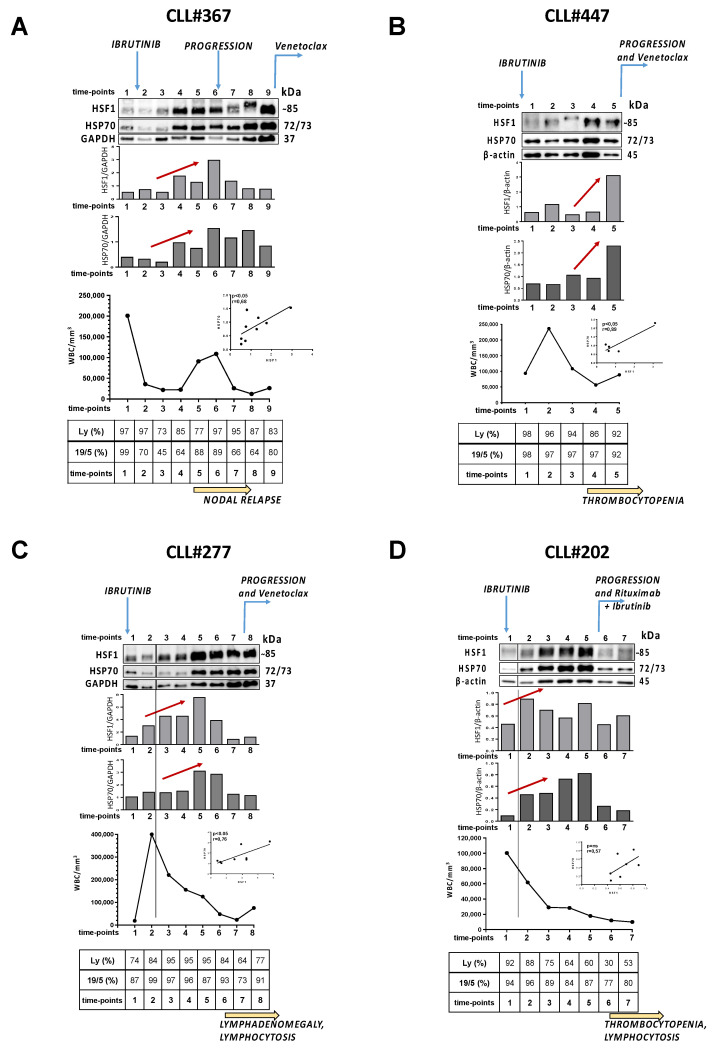
HSP70 and HSF1 expression levels remain elevated in patients who fail treatment with Ibrutinib. Data are related to patient #367 ((**A**), time-points: 1 to 9), #447 ((**B**), time-points: 1 to 5), #277 ((**C**), time-points: 1 to 8), #202 ((**D**), time-points: 1 to 7). From top to bottom: analysis of HSF1, HSP70 and GAPDH or β-actin by Western blotting; densitometric analysis of HSF1/GAPDH or β-actin and HSP70/GAPDH or β-actin ratios at the different time-points; patient’s white blood cell count; percentage of lymphocytes/total white blood cells; percentage of CD19^+^/CD5^+^ lymphocytes/total number of lymphocytes. At the top of all sections, blue arrows indicate the start of treatment with ibrutinib, the start of progression and the start of another treatment (e.g., venetoclax, anti-Bcl2). At the bottom, yellow arrows indicate the onset of clinical disease progression (e.g., lymph node recurrence, thrombocytopenia, etc.). Inside the figure, red arrows indicate an increase in the expression of HSP70 and HSF1. Linear correlation graphs are related to HSP70 expression level vs. HSF1 expression levels at each time-point (*p* < 0.05 for A, B and C; Pearson’s correlation). WBC: white blood cells.

**Figure 4:**
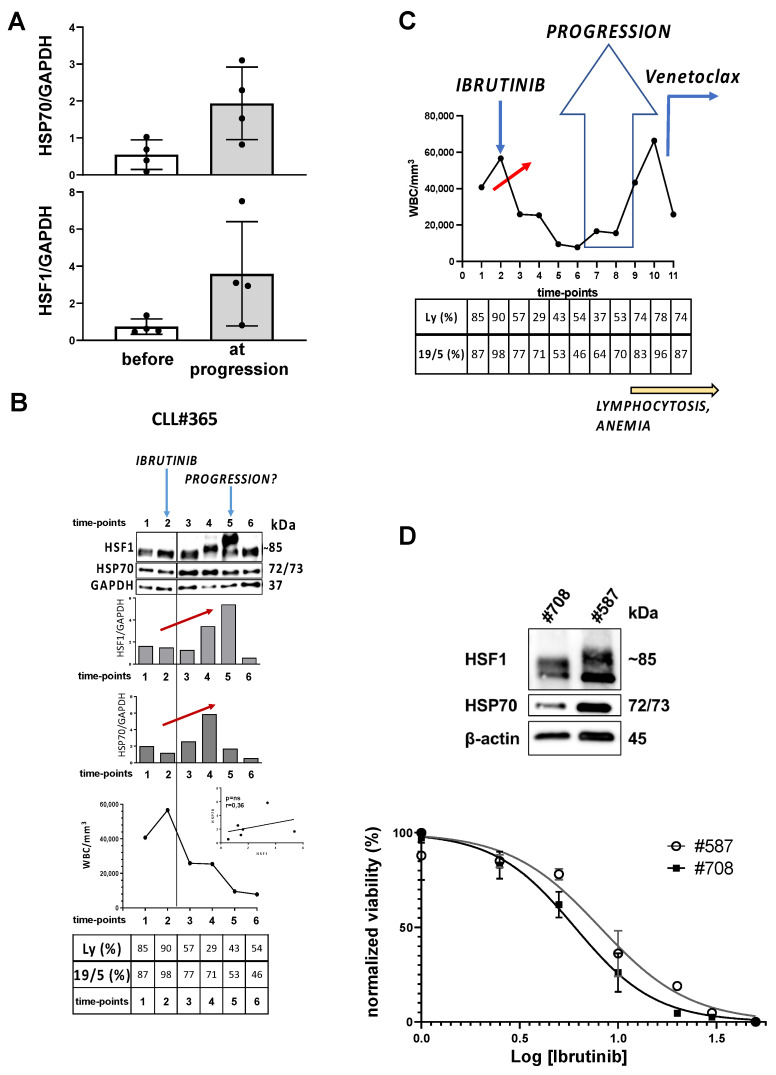
HSP70 and HSF1 expression levels predict clinical progression during ibrutinib treatment. (**A**). Histograms report the densitometric analysis of HSF1/GAPDH and HSP70/GAPDH ratios obtained after Western blotting analysis at the beginning of the treatment (before) and at a time-point identified as clinical progression (at progression). Analysis had been performed in four patients (#367, #447, #277, #202) who showed progression during ibrutinib assumption in vivo. (**B**). Data are related to patient #365 (time-points: 1 to 6). From top to bottom: analysis of HSF1, HSP70 and GAPDH by Western blotting; densitometric analysis of HSF1/GAPDH and HSP70/GAPDH ratios at the different time-points; patient’s white blood cell count; percentage of lymphocytes/total white blood cells; percentage of CD19^+^/CD5^+^ lymphocytes/total number of lymphocytes. At the top of the sections, blue arrows indicate the start of treatment with ibrutinib and the start of the hypothetic progression. Inside the figure, red arrows indicate the increase in the expression of HSP70 and HSF1. Linear correlation graphs are related to HSP70 expression level vs. HSF1 expression levels at each time-point. WBC: white blood cells. (**C**). Data are related to patient #365 and proposed again in this figure with a wider time-range. From top to bottom: patient’s white blood cell count, percentage of lymphocytes/total white blood cells and percentage of CD19^+^/CD5^+^ lymphocytes/total number of lymphocytes at different time-points. At the top of the sections, blue arrows indicate the start of treatment with ibrutinib and the start of another treatment (i.e., venetoclax, anti-Bcl2). The big blue arrow represents the period in which the progression was confirmed. Inside the figure, red arrow indicates the increase in the expression of HSP70 and HSF1. At the bottom, yellow arrow indicates the onset of clinical disease progression (e.g., lymphocytosis and anemia). WBC: white blood cells. (**D**). Freshly isolated leukemic B cells from therapy-free CLL patients (#708 and #587) were subjected to cell lysis and western blotting and then immunostained with anti-HSP70, anti-HSF1 and anti-β-Actin as loading control. In the graph below, dose-response curves for IC_50_ calculation. 5 × 10^5^ cells have been treated with increasing doses of ibrutinib and, after 72 h, cell viability has been assessed by flow cytometry and plotted.

We analyzed HSP70 and HSF1 expression in patients who failed ibrutinib treatment. These patients were initially sensitive to ibrutinib therapy, and showed a measurable tumor regression, but then they had progressive disease (defined as having an increasing number of lymphocytes increase, or the enlargement of lymph nodes), with a concomitant increase in the expression of the two proteins taken into account. HSP70 and HSF1 levels were determined by western blotting in freshly isolated B cells from patients #367, #447, #277, and #202 who underwent an ibrutinib-containing regimen. Cells have been analyzed during therapy at different time-points (every 3 or 4 months), even after the documented relapse. Figure 3A–D summarize the data obtained for each patient. Protein expression of HSP70 and HSF1 and the relative densitometric analysis have been reported; red arrows represent the increase in expression of HSP70 and HSF1 in a time ranging from the beginning of the observation and the progression. The figure also shows the trend of the white blood cell count (WBC) and the percentage of lymphocytes in the peripheral blood of each patient, and the number of neoplastic CD19^+^/CD5^+^ cells. It is significant how the increase in the expression of HSP70 and HSF1 manifests around the clinical progression of the disease: the quantitative growth of the two proteins records a significant peak before the clinical relapse (assessed as nodal relapse, lymphocytosis, etc.). Of note, an upward shift of the band relative to HSF1 is noticeable in several lanes, perhaps due to the activating phosphorylation of the protein at Ser326 [10,16]. Furthermore, the increase in HSP70 and HSF1 levels indicates, as already demonstrated [10], that the two proteins are positively correlated. In the considered range of treatment, we assist to an increase of HSP70 from 0.55 ± 0.39 to 1.93 ± 0.98 and of HSF1 from 0.73 ± 0.41 to 3.59 ± 2.81 with a fold induction of 4.78 ± 2.73 and 4.49 ± 1.82 for HSP70 and HSF1, respectively (Figure 4A).

### 2.4. Increased Expressions of HSP70 and HSF1 Are Markers of Disease Progression

In our study, we came across the case of patient #365, still responsive to ibrutinib and in good general condition, with a low WBC count, who exhibited higher HSP70 and HSF1 levels than other therapy-sensitive patients (Figure 4B), which was a dubious trend. Moreover, in this case, a shift relative to HSF1 is appreciable at time-points 4 and 5, maybe due to HSF1 constitutive activation at Ser326 [10,16]. Based on the data so far obtained, we wondered if a high expression of HSP70 and HSF1 represents the phase immediately prior to the clinical progression of the disease. Indeed, this patient after 60 months has become unresponsive to ibrutinib. This was clinically highlighted by lymphocytosis and anemia, so that this patient has been initiated to a salvage treatment based on venetoclax, as reported in Figure 4C. Another evidence supporting the hypothesis that HSP70 and HSF1 may be markers of disease progression is reported in Figure 4D; here, HSP70 and HSF1 overexpression is associated with a major resistance to ibrutinib, as assessed by IC_50_ calculation in CLL B cells from therapy free patients. For the two samples analyzed, patient #587 with a higher IC_50_ for ibrutinib (=8.092 μM) had also a higher expression of both HSP70 and HSF1 than patient #708, presenting a lower IC_50_ (=6.040 μM; *p* < 0.0001 vs. #587, extra-sum-of-square F test).

### 2.5. HSP70/HSF1 Axis Inhibitors Induce In Vitro Cell Death in Neoplastic Cells from Patients Who Relapsed during Ibrutinib

**Figure 5:**
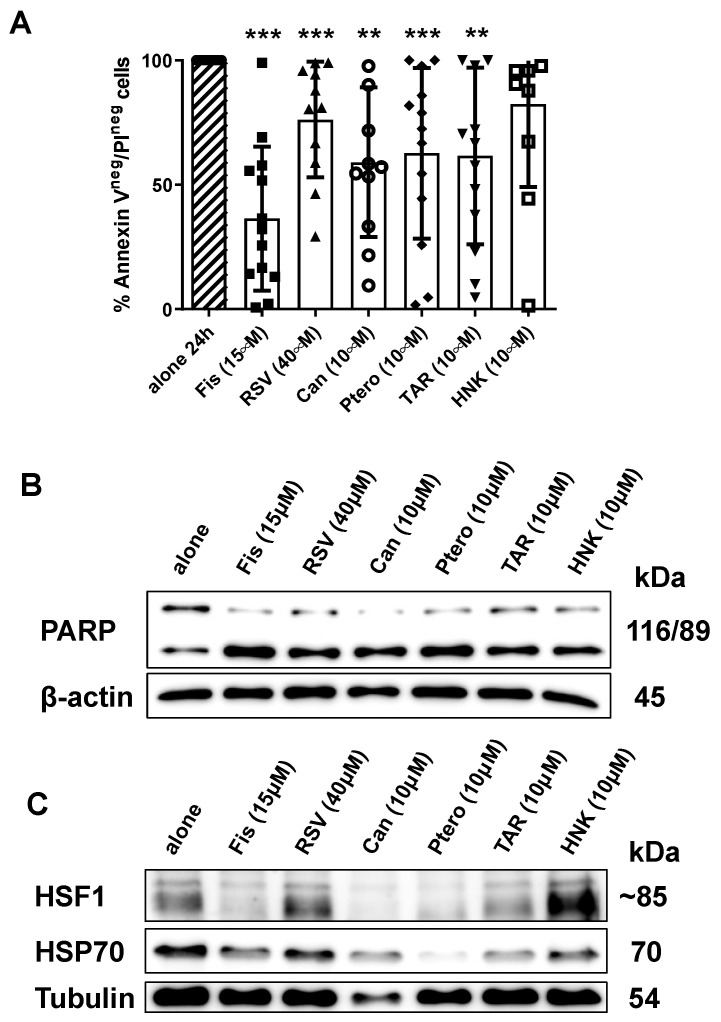
Resveratrol and related compounds induce apoptosis in leukemic B cells from patients who failed Ibrutinib treatment. (**A**). Leukemic B cells from CLL patients failing Ibrutinib treatment were cultured alone or in the presence of 15 µM Fis, 40 µM RSV, 10 µM Can, 10 µM Ptero, 10 µM TAR and 10 µM Hnk and cell apoptosis was analyzed by annexin V/PI flow cytometric test. Histograms report the percentage of Annexin V^neg^/PI^neg^ cells after 24 h treatment and the treated conditions were normalized on the untreated one. Data are reported as mean ± SD (** *p* < 0.01 and *** *p* < 0.001, paired Student’s *t* test; *n* = 13). (**B**). Lysates obtained from CLL B cells from patients failing Ibrutinib treatment cultured alone or with 10 µM Fis, 40 µM RSV, 10 µM Can, 10 µM Ptero, 10 µM TAR and 10 µM Hnk were analyzed by immunostaining with anti-PARP for apoptosis detection and anti-β-actin as loading control. Western blotting is representative of all the cases analyzed (*n* = 13). (**C**). Lysates obtained from CLL B cells form patients failing Ibrutinib treatment cultured alone or with 10 µM Fis, 40 µM RSV, 10 µM Can, 10 µM Ptero, 10 µM TAR and 10 µM Hnk were analyzed by immunostaining with anti-HSF1, anti-HSP70 and anti-β-actin or tubulin as loading control. Western blotting is representative of all the cases analyzed (*n* = 13). Fis: Fisetin; RSV: Resveratrol; Can: Cantharidin; Ptero: Pterostilbene; TAR: Triacetyl Resveratrol; Hnk: Honokiol. PI: Propidium Iodide.

Considering that (i) the examined molecules (i.e., HSP70 and HSF1) are overexpressed in CLL cells, particularly at the beginning of the clinical progression in those patients failing ibrutinib-containing regimen and that (ii) resveratrol and its associated molecules (i.e., pterostilbene, triacetyl-resveratrol and honokiol) or anyway HSP70 (i.e., pifthrin) [9,28] or HSF1 (i.e., fisetin) [10,29] inhibitors have been proven to induce cell death by inhibiting HSP70/HSF1 signaling; we hypothesized their effectiveness in inducing apoptosis in cells from ibrutinib-resistant patients. To test this hypothesis, we cultured cells from ibrutinib-resistant patients, whose characteristics are reported in Table 1, with the above-mentioned inhibitors. Apoptosis induction has been investigated by annexin V/propidium iodide staining using flow cytometry test and by Western blot analysis of cleave-PARP. After 24 h of incubation, we observed a significant reduction of cell viability in treated vs. untreated cells. In particular, the quote of viable cells was 36.46% ± 28.92% (*p* < 0.001, paired Student’s *t* test; viable cells percentage of the treated condition was normalized on the untreated one) after 15 µM fisetin, 76.21% ± 23.25% (*p* < 0.001) after 40 µM resveratrol, 61.61% ± 35.45% (*p* < 0.01) after 10µM triacetyl-resveratrol, 62.67% ± 34.29% (*p* < 0.001) after 10 µM pterostilbene. Another compound, named cantharidin, that induces cell death through the inhibition of HSP70 expression by blocking HSF1 binding to promoters, induces significant apoptosis in ibrutinib-resistant cells (*p* < 0.01) at 10 µM. As for 10µM honokiol, the reduction in cell viability was not significant (Figure 5A). For all the tested molecules, apoptosis induction has been confirmed by the increase of the cleaved form of PARP (Figure 5B). Finally, for all the examined conditions, we evaluated whether or not there was a reduction in the expression of HSF1 and HSP70 compared to control cases incubated with medium alone. Both proteins were found to be down-modulated after treatment with fisetin, cantharidin, pterostilbene and triacetyl-resveratrol with respect to the untreated condition, as shown in Figure 5C.

## 3. Discussion

In this work, we demonstrated an involvement of the HSP70/HSF1 axis in ibrutinib-resistance in chronic lymphocytic leukemia (CLL), and the effect of the inhibition of this axis as a basis for identifying new strategies in CLL treatment. CLL has an extremely heterogeneous clinical course, which reflects the heterogeneity of its pathogenetic alterations. This disease is currently incurable, although drug research is making huge progresses. In recent years, new molecular targeted therapies have been developed, including ibrutinib, a Btk inhibitor, which has changed the management and clinical history of patients with multi-treated CLL, or with a very poor prognostication [30,31,32]. Despite ibrutinib being extremely effective, discontinuation of therapy is present, often due to the development of adverse events and resistance to treatment, which manifest with evidence of clinical and biochemical progression during therapy [33]. In 80% of patients failing ibrutinib, the development of resistance is due to mutations affecting Btk and/or PLCγ [34], the downstream effector of Btk pathway, which make the drug no longer capable of irreversibly inhibiting Btk pathway [8]; in the remaining 20% cases, the pathological mechanism of resistance is still unknown, and therefore research in this field is very active. A broader understanding of the pathways that regulate signaling in CLL would allow the development of alternative strategies to overcome the resistance of a drug which, data at hand, has proven to be extremely effective. In this perspective, we focused on two proteins, HSP70 (heat shock protein of 70kDa) and its transcription factor HSF1 (heat shock factor 1), since they are implicated in various stages of carcinogenesis, such as the promotion of cell proliferation, resistance to apoptosis, neo-angiogenesis, metastasis and, above all, resistance to radio- and chemo-therapy [14,35]. Moreover, post-translational modifications regulate and modulate HSP70 and HSF1 activity in normal and cancer cells [16,36]. In the hematology field, Pocaly et al. proved that HSP70 overexpression is associated with drug-resistance to Imatinib, a small molecule used for chronic myeloid leukemia (CML) treatment [37]. Moreover, Steiner and colleagues highlighted how high levels of HSP70 are associated with a poor prognosis in patients with acute myeloid leukemia (AML) [38]. Herein, our aim was to evaluate HSP70/HSF1 axis involvement in ibrutinib-resistance in CLL and the effect of the inhibition of this axis as a strategy to bypass ibrutinib-resistance. We previously demonstrated that both proteins (HSP70 and HSF1) are overexpressed and activated in B cells from therapy-free patients with respect to normal B cells, particularly in poor prognosis patients. Furthermore, the levels of both proteins consensually decrease in patients responsive to chemo-immunotherapy in vivo, while they remain stable or increase in those subjects who progress during therapy [10]. Considering the importance of HSP70 and HSF1 in CLL clinical course, we evaluated whether HSP70/HSF1 inhibitors were able to induce in vitro apoptosis of CLL cells. Although the molecules we used (pifithrin-μ [28], VER-155008 [39], zafirlukast [40]) have proven to be effective in determining cell death in vitro, as well as other molecules described in the literature [11]; studies with direct inhibitors of HSP70 have not been deepened, since the direct inhibition of HSP70 collaterally induces the expression of other chaperones that are toxic to the cell. With the other approach, which is the targeting of HSF1, we demonstrated that fisetin, by inhibiting the binding of HSF1 to HSP70 promoter, determined apoptosis in CLL cells. To better understand HSP70 and HSF1 regulation, we studied their RAS-dependent activation pathway in CLL (as described in Figure 1A), pointing out how high levels of HSP70 could be the result of HSF1 hyperactivation due, at least in part, to both the activation of PI3K/AKT/mTOR signal pathway and the inhibition of the RAF/MEK/ERK pathway [10]. With this as a background, given the interaction of the signal pathways mediated by RAS with HSP70/HSF1 and supported by the study of Mustafi et al. on CML cells [17], we used resveratrol, a natural polyphenol equipped with anti-cancer properties through the modulation of different kinases (e.g., Akt and Erk1/2) and transcription factors (e.g., Nf-kB [41]). This molecule induced the death of CLL cells by reducing the levels of HSP70 and HSF1, also confirming the HSF1 regulation model previously developed (Figure 1A). However, since this molecule has low bioavailability and poor potency, we performed the same experiments with other inhibitors with a structure and action like resveratrol, namely pterostilbene [42], triacetyl resveratrol [43] and honokiol [44], already known for their anti-cancer properties. All these molecules were shown to be significantly effective in inducing apoptosis in CLL cells of therapy-free patients at a concentration lower than that used for resveratrol. This approach that indirectly down-modulates HSP70 aims at reducing the unpredictable off-target effects that the direct inhibition of HSP70 protein has shown. Since HSP70 overexpression is co-responsible for the reduced response to chemotherapy [6], allowing cancer cell survival in a “stressful” environment, and strengthened by our previous study on the correlation between therapeutic response after chemo-immunotherapy and HSP70 and HSF1 levels, we evaluated the expression of these two proteins in CLL patients who failed treatment with ibrutinib, experiencing disease progression during its assumption. We found a correlation between HSP70/HSF1 levels and response to therapy, demonstrating previously a reduction in responsive patients [10], and then an increase in non-responsive patients at the clinical progression of the disease. This leads us to hypothesize a role for HSP70 and HSF1 as markers for detecting drug resistance early, in part confirmed by patient #365, whose HSP70 and HSF1 levels were elevated, although he did not yet show clinical signs of progression, that has then been experienced after 60 months of ibrutinib assumption. Moreover, patients who exhibit a “resistance” to ibrutinib in vitro, in terms of higher IC_50_ to this drug, also present higher levels of HSP70 and HSF1. Considering the data so far obtained, that is: (i) HSP70/HSF1 overexpression in CLL cells, especially at the beginning of disease progression in those patients who failed ibrutinib, and (ii) the efficacy of HSP70 or HSF1 inhibitors in inducing death in CLL cells, we hypothesized an efficacy of these molecules in inducing apoptosis, even in cells from patients resistant to ibrutinib. Despite the small number of cases, our data are encouraging, since the percentage of viable cells were significantly reduced, both with the direct inhibitors of HSF1 (i.e., fisetin and cantharidin) and resveratrol-associated molecules. Furthermore, we highlighted a correlation between cell mortality and HSP70/HSF1 levels; that is, a reduction in the expression of HSP70 and HSF1 in apoptotic cells, thus pointing out how the inhibitory action on this axis determines apoptosis in leukemic cells. In conclusion, although further verifications must be made, from the collected results, we can establish a role for the HSP70/HSF1 axis and, probably, for other proteins associated with it (e.g., BAG3 [45,46]) in the phenomena of drug-resistance to ibrutinib. Furthermore, thanks to HSP70/HSF1 axis regulatory model (Figure 1A), we can state that the indirect strategy of the inhibition of HSF1 and HSP70 realized by resveratrol-associated molecules is effective in inducing cell death in ibrutinib-resistant cells. Our work is supported by literature where resveratrol and related stilbenoids have been demonstrated to induce apoptosis in human myeloid leukemia cells [47]. These molecules are easily editable, as has been demonstrated in Inoue et al. [48], where an in-house chemical library bearing resveratrol building blocks was screened for cytotoxic effects in several leukemia and cancer cell lines and their anti-cancer drug-resistant derived sublines. Moreover, it is reported that resveratrol affects the proliferation and apoptosis of human acute promyelocytic leukemia cells by regulating the PTEN/PI3K/AKT pathway [49]. For all these reasons, resveratrol and its analogs should no longer be considered only as “natural remedies” or “dietary supplements”, but real kinase inhibitors to be exploited alone or in combination with hematological tumors. Finally, the dissection of the HSP70/HSF1 axis will contribute to defining the biology and the progression of CLL.

## 4. Materials and Methods

### 4.1. Patients and Cell Separation

We overall obtained peripheral blood from 35 untreated patients who satisfied standard criteria for CLL. Written informed consent was obtained according to the Declaration of Helsinki, and ethical approval was obtained from the local ethic committee. Peripheral blood mononuclear cells (PBMC) of the patients were isolated by density-gradient centrifugation over lymphoprep (STEMCELL Technologies; Vancouver, Canada). Where necessary, further purifications, including alternately, the use of the “Human B cell enrichment cocktail” (STEMCELL Technologies), or the use of the separation method of sheep red blood cells (SRBC from Neomed; Milan, Italy) were performed. The second method exploits the unique ability of T cells to bind to and to form rosettes with SRBC. Briefly, aliquots of 25 × 10^6^ CLL PBMCs were incubated on ice with 1mL of SRBC pre-treated with neuraminidase. After incubation, the cell suspension was centrifuged, the supernatant was discarded, and the pellet was resuspended in saline, and then subjected to a further density-gradient centrifugation over Lymphoprep. After centrifugation, T cells and SRBC were found in the pellet, while the “non-T” fraction (B-cells in the case of CLL) was in the interphase. Finally, all the samples utilized had CD19^+^/CD5^+^ B-cell content greater than 95%. Untreated peripheral blood B cells were isolated from PBMCs of 4 normal donors, representative of the adult healthy population, using the “Human B cell enrichment cocktail”. The purity of the obtained B cells was at least 95% (CD19^+^), as assessed by flow cytometry. As regards experiments with B cells obtained from patients in therapy, we used B cells obtained from patients with CLL resistant to ibrutinib before and during therapy (Table 1). Protocol “Ibrutinib”: 420mg ibrutinib once a day till progression.

### 4.2. Culture Conditions, IC_50_ Calculation and Apoptosis Detection by Flow Cytometry

For experiments with inhibitors, cells (2 × 10^6^/mL) were cultured as previously described [50], and the inhibitors used were as follows: 40 μM of resveratrol (RSV); 10, 20 and 30 μM pterostilbene (Ptero); 10, 50 and 100 μM triacetyl resveratrol (TAR); 5, 10 and 20 μM honokiol (Hnk), 10 μM cantharidin (Can) and 10 μM fisetin (Fis); for experiments, according to a time-course evaluation, we chose the 24 h time point. Concentrations for the different inhibitors were chosen according to literature data and preliminary experiments.

For IC_50_ calculation, CLL cells were cultured in triplicate with increasing doses of ibrutinib (0, 1, 2.5, 5, 10, 20, 30 and 50 μM). Cell viability after 72 h was analyzed by flow cytometry considering as live cells those identified at the morphological gate. The obtained percentages at each dose were used to determine the concentration of the inhibitors at which a 50% of reduction in cell viability was observed by the generation of a dose-response curve by Prism 9 (GraphPad Software Inc.; La Jolla, CA, USA).

Apoptosis was assessed using the Annexin V Apoptosis Detection Kit (Valter Occhiena S.r.l., Turin, Italy). Briefly, aliquots of 5 × 10^5^ cells were harvested, washed, and incubated for 10min in the dark at room temperature with 100 μL of binding buffer, 3 μL of annexin V-fluorescein isothicyanate (FITC), and 3 mL of propidium iodide (PI). Then, another 100 μL of binding buffer was added before the acquisition at the flow cytometer (BD FACSCanto™ II Cell Analyzer; BD Biosciences, San Jose, CA, USA).

### 4.3. Western Blotting Analysis

Samples were prepared by cell lysis, as previously described [50]. The antibodies used were as follows: anti-PARP (Cell Signalling Technology Inc.; Danvers, MA, USA), anti-HSF1 (Enzo Life Sciences, Inc.; Farmingdale, NY, USA), anti-HSP70, anti-GAPDH (Abcam; Cambridge, UK), anti-tubulin and anti-β-actin (Sigma-Aldrich; Saint Louis, MO, USA). Original Western blotting is displayed in the Supplementary Materials (Figure S2).

### 4.4. Statistical Analysis

Statistical analyses were performed using Prism 9 by Student’s *t* test, paired Student’s *t* test and Wilcoxon test. Data were considered statistically significant when *p* values were <0.05.

## 5. Conclusions

In several tumors, HSP70 and HSF1 overexpression is one of the causes of low therapy response, allowing cancer cells to escape apoptosis and survive despite stressing conditions. Our findings suggest an involvement of HSP70/HSF1 axis in controlling pharmacological resistance to ibrutinib in CLL cells, since their inhibition is effective in inducing in vitro apoptosis in cells from ibrutinib refractory patients. For this reason, the use of HSP70 and HSF1 inhibitors could represent a novel approach (alone or in combination) to overcome ibrutinib resistance in those patients who relapsed after this type of treatment; moreover, these proteins could be used as a marker to find early relapse in patients. Considering the role played by ibrutinib in the management of different non-Hodgkin Lymphomas (i.e., MZL, MCL, DLBCL, WM), HSP70/HSF1 axis inhibition would be of clinical relevance not only in CLL, but also in other B-lymphoproliferative diseases.

## Figures and Tables

**Table 1:** Ibrutinib-resistant patients’ characteristics.

CLL	M/F	Age	IGHV ^1^	FISH	TP53 ^2^	Th before Ibr ^3^	Ibr (Time, Months) ^4^	Cause of Interrumption	Th after Ibr ^5^	Transformation
#23	M	69	U	17p; 12+	M	Y	3		Idelalisib, Venetoclax	PLL
#202	M	78	M	13q	WT	N	72	thrombocytopenia, lymphocytosis	Rituximab + Ibrutinib	
#234	F	69	U	13q borderline	nd	Y	18		Venetoclax	Richter
#265	F	79	U	13q	M	Y	12		Venetoclax	
#365	M	81	U	17p	nd	Y	60	lymphocytosis, anemia	Venetoclax	
#366	F	74	M	12+; 13q	M	Y	30	nodal relapse	Venetoclax, Idelalisib	Richter
#447	F	71	U	normal	M	Y	24	thrombocytopenia	Venetoclax, Idelalisib	
#480	F	82	U	13q	nd	Y	12			
#565	M	64	U	11q	WT	Y	30		Venetoclax	
#627	F	66		17p; 13q	nd	N	5		Venetoclax	

^1^ IGHV: immunoglobulin heavy-chain variable region gene; U: unmutated, M: mutated-defined as having a frequency of mutations greater than 2% from germline VH sequence. ^2^ TP53 gene sequencing and analysis were performed according to ERIC guidelines [ref. Malcikova J., 2018 Leukemia 32, 1070–1080] M: mutated, WT: Wild Type. ^3^ Th before Ibr: if patient has received other therapies before Ibr. ^4^ duration of Ibr treatment before progression (months). ^5^ salvage treatment after Ibr. M/F: male/female. FISH: fluorescence in situ hybridization. Th: therapy. Ibr: Ibrutinib. Y: Yes; N: No. PLL: Prolymphocytic Leukemia.

## Data Availability

The data presented in this study are available in this article (and Supplementary Materials).

## Supplementary Materials

The following are available online at https://www.mdpi.com/article/10.3390/cancers13215453/s1, Figure S1: Effect of Pterostilbene, Triacetyl Resveratrol and Honokiol in normal B cells. Figure S2: Whole blots relative to the Western Blotting analyses.
