# Supplementary material for: Targeting of HSP70/HSF1 Axis Abrogates In Vitro Ibrutinib-Resistance in Chronic Lymphocytic Leukemia
Source: Cancers (Basel). 2021 Oct 29;13(21):5453. doi: 10.3390/cancers13215453 (PMC8582437; doi:10.3390/cancers13215453)
Supplement: Supplementary file 1 [file cancers-13-05453-s001.zip › cancers-1375921-supplementary.pdf]

Supplementary material

Targeting of HSP70/HSF1 Axis Abrogates *in vitro* Ibrutinib-Resistance in Chronic Lymphocytic Leukemia.

by

Frezzato Federica, Visentin Andrea, Severin Filippo, Pizzo Serena, Ruggeri Edoardo, Leonardo Martinello, Elisa Pagnin, Trimarco Valentina, Tonini Alessia, Carraro Samuela, Stefano Pravato, Silvia Imbergamo, Francesco Piazza, Anna Maria Brunati, Facco Monica, Trentin Livio.

Figure S1. Effect of Pterostilbene, Triacetyl Resveratrol and Honokiol in normal B cells.

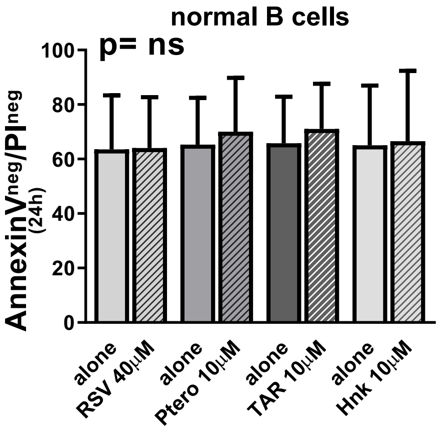

Purified B cells from healthy donors were cultured alone or in the presence of 40μM RSV, 10μM Ptero, 10μM TAR and 10μM Hnk; cell apoptosis was analyzed by annexin V-PI flow cytometric test. Histograms report the percentage of Annexin V^{neg}/PI^{neg} cells after 24h treatment. Data are reported as mean±SD (p=ns, treated *vs* untreated conditions, Wilcoxon test; n= 4).

Figure S2. Whole blots relative to the Western Blotting analyses.

Figure 1C

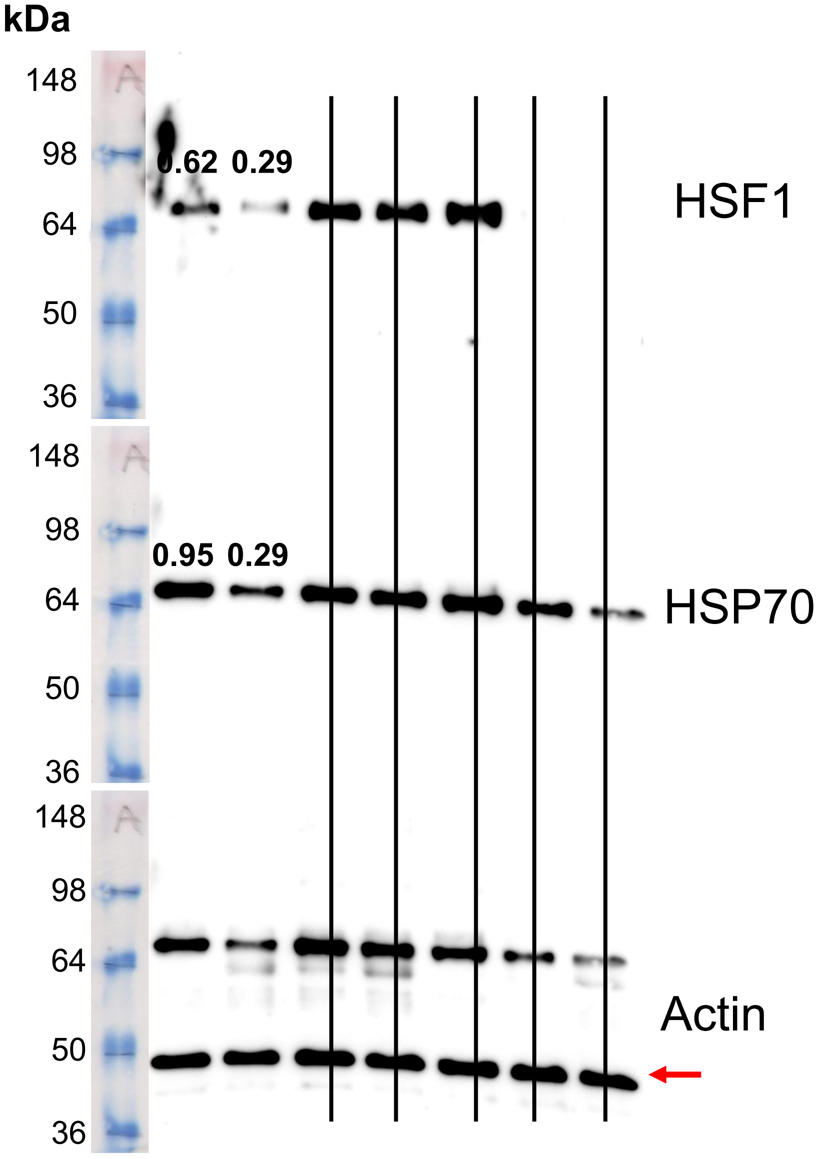

Deleted lanes are related to experiments that don't deal with the present manuscript.

Figure 2E

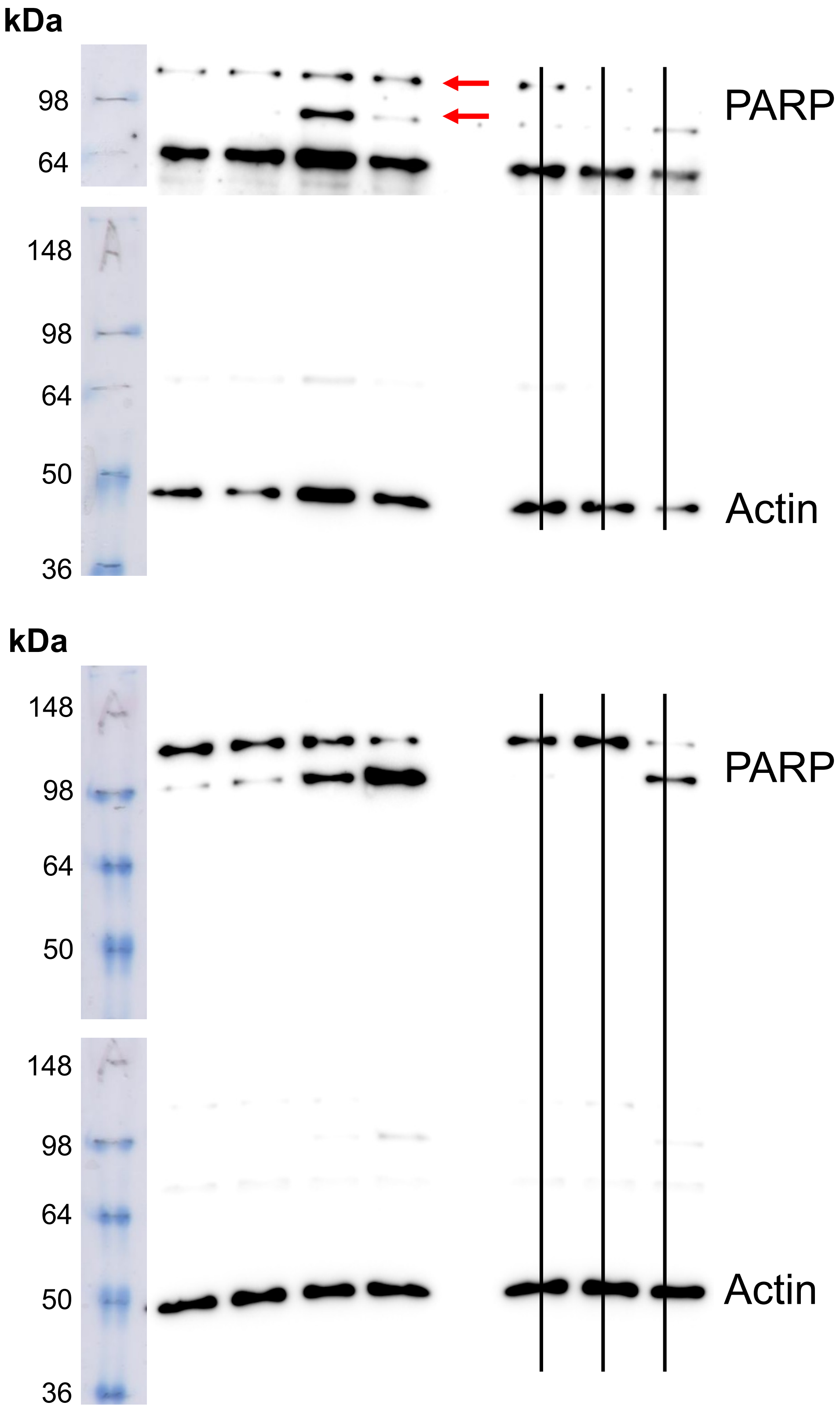

Deleted lanes are related to experiments that don't deal with the present manuscript.

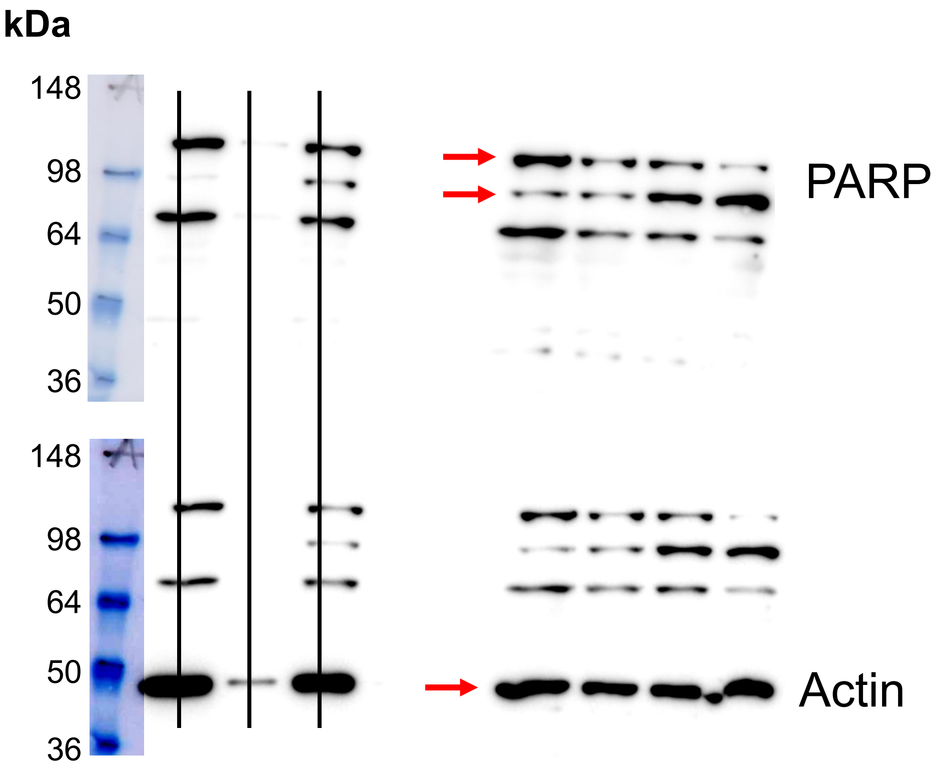

Deleted lanes are related to experiments that don't deal with the present manuscript.

Figure 2F

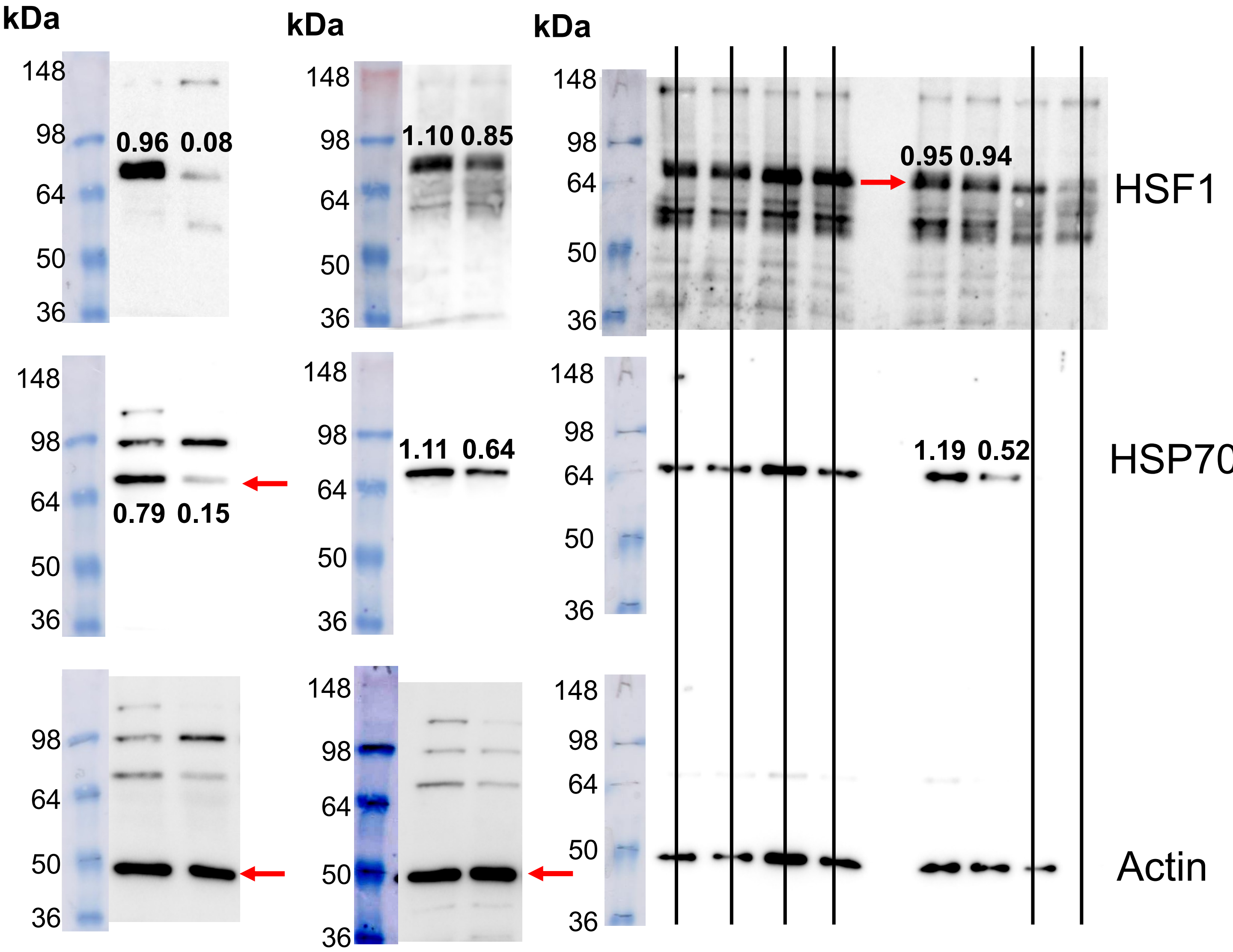

Deleted lanes are related to experiments that don't deal with the present manuscript.

Figure 3A

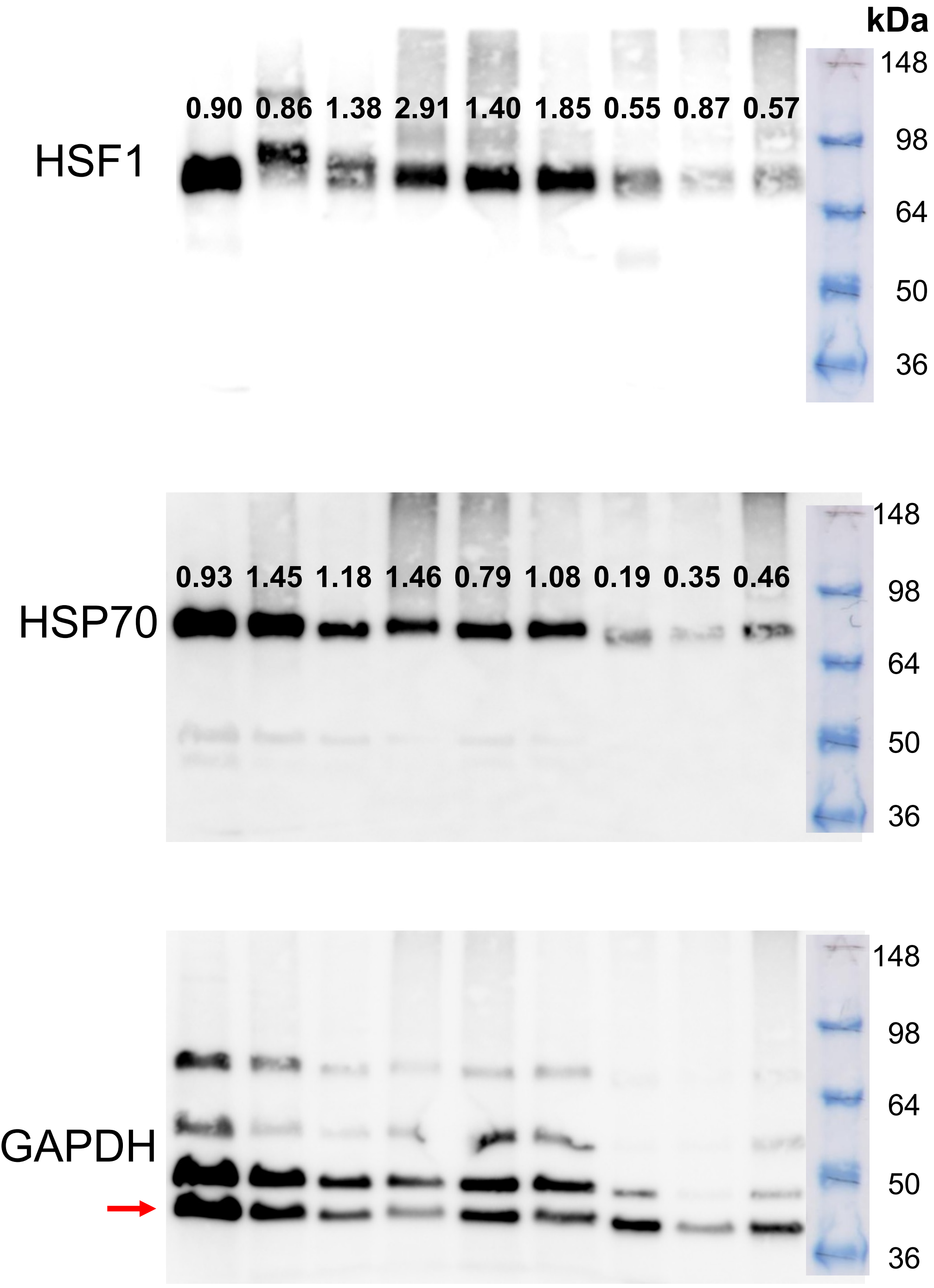

In the manuscript, these blots are flipped orizzontally.

Figure 3B

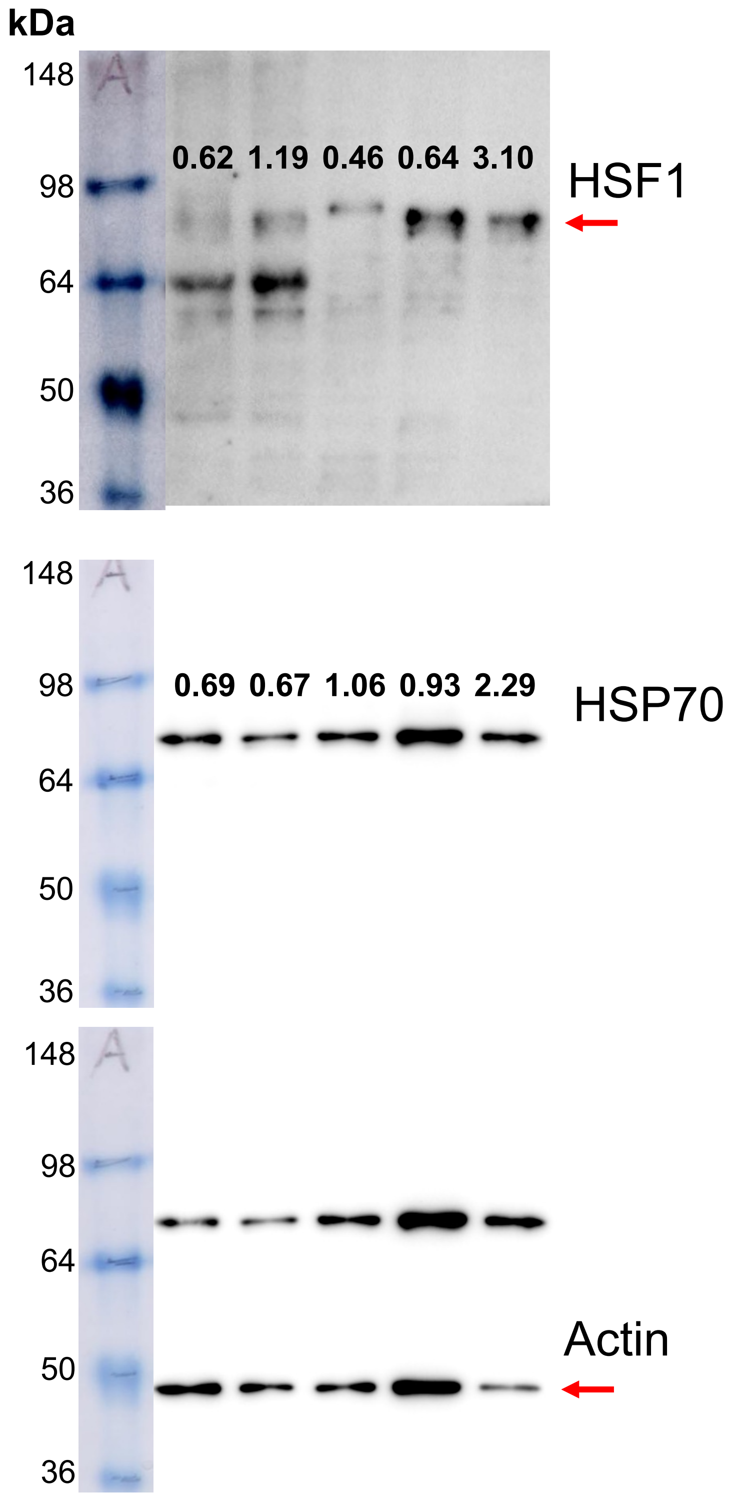
kDa
148
98
64
50
36
0.62 1.19 0.46 0.64 3.10
HSF1
148
98
64
50
36
0.69 0.67 1.06 0.93 2.29
HSP70
148
98
64
50
36
Actin

Figure 3C

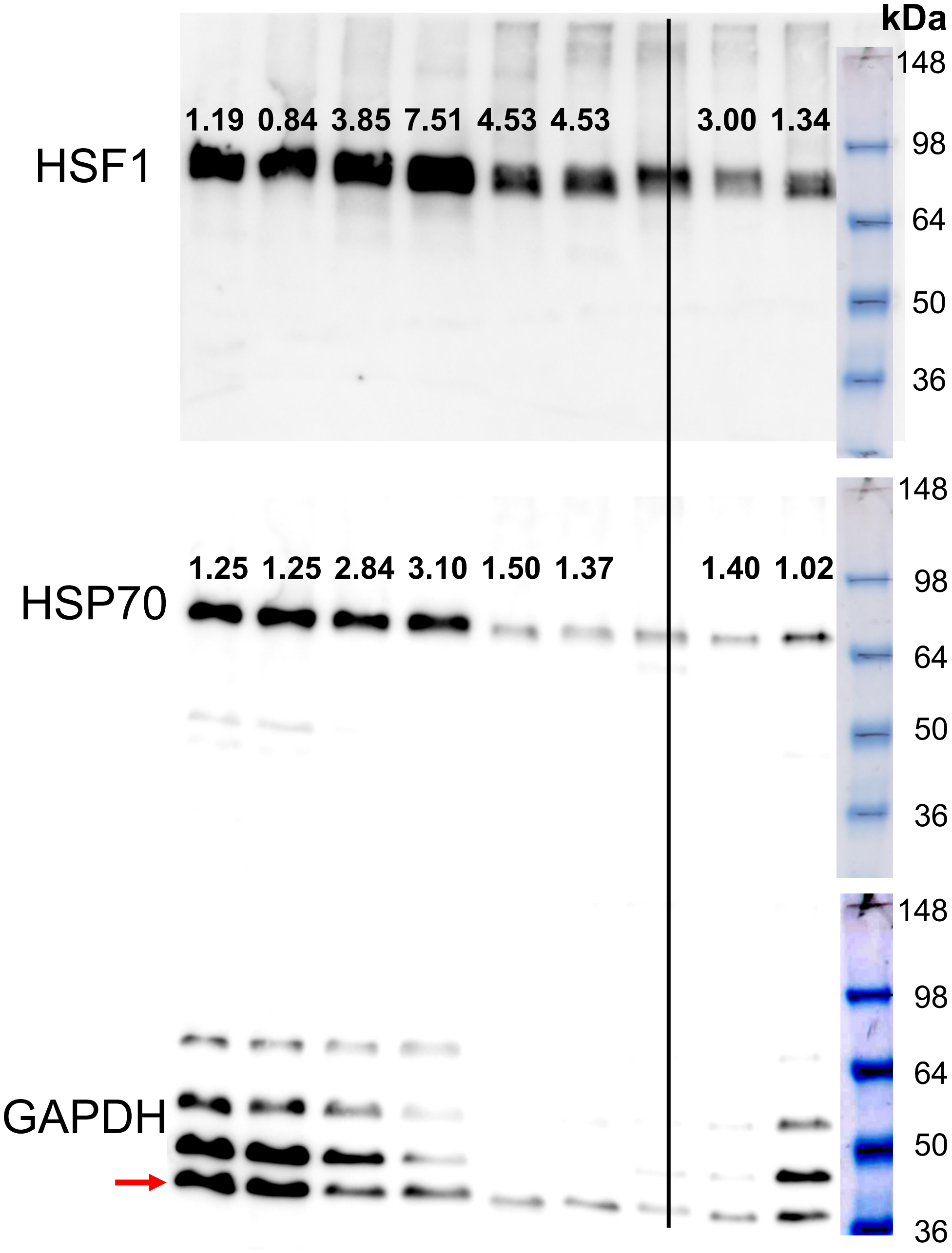

In the manuscript, these blots are flipped orizzontally and deleted lane represents a loading error and were not included in the manuscript.

Figure 3D

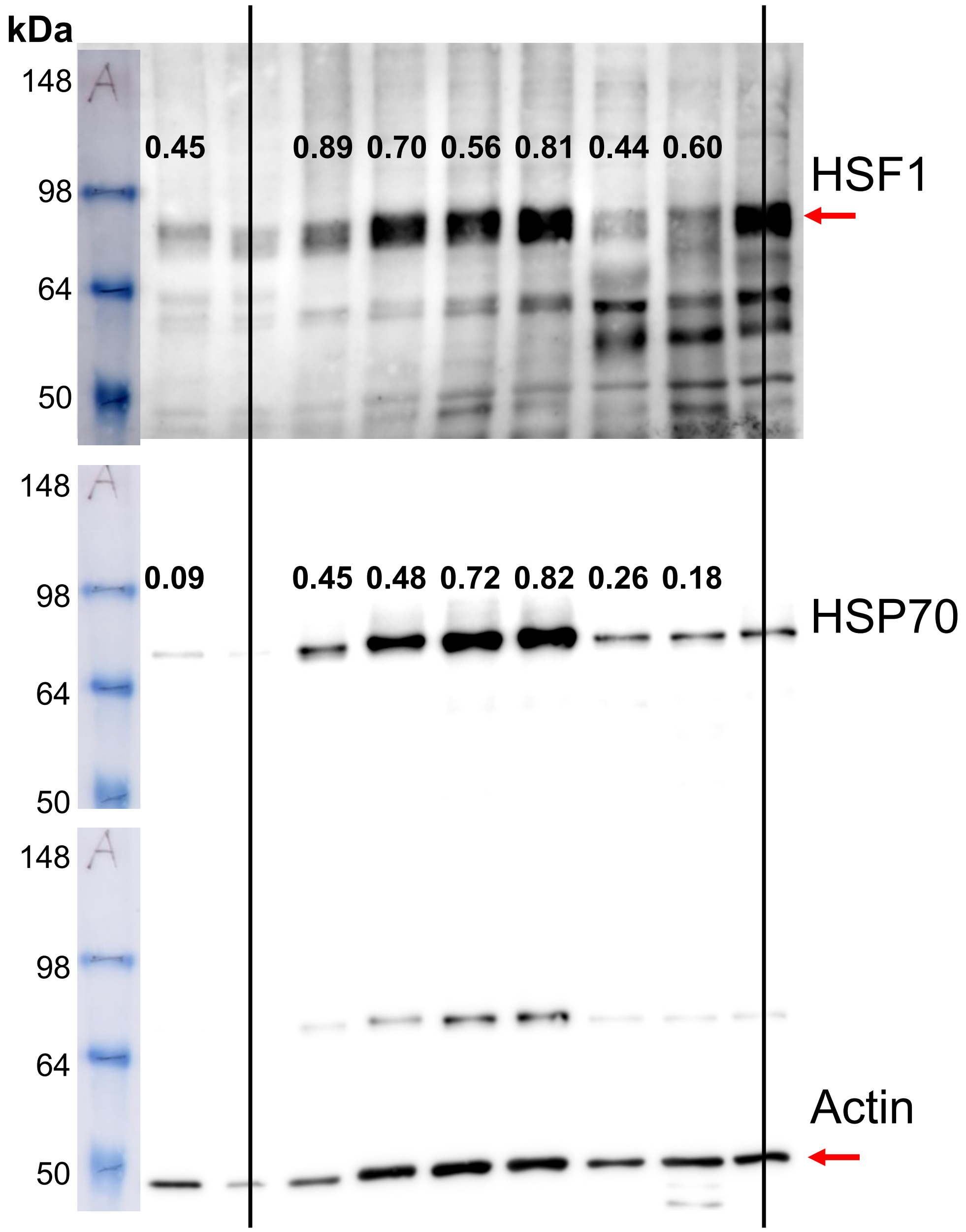

Deleted lanes represent a loading error and were not included in the manuscript.

Figure 4B

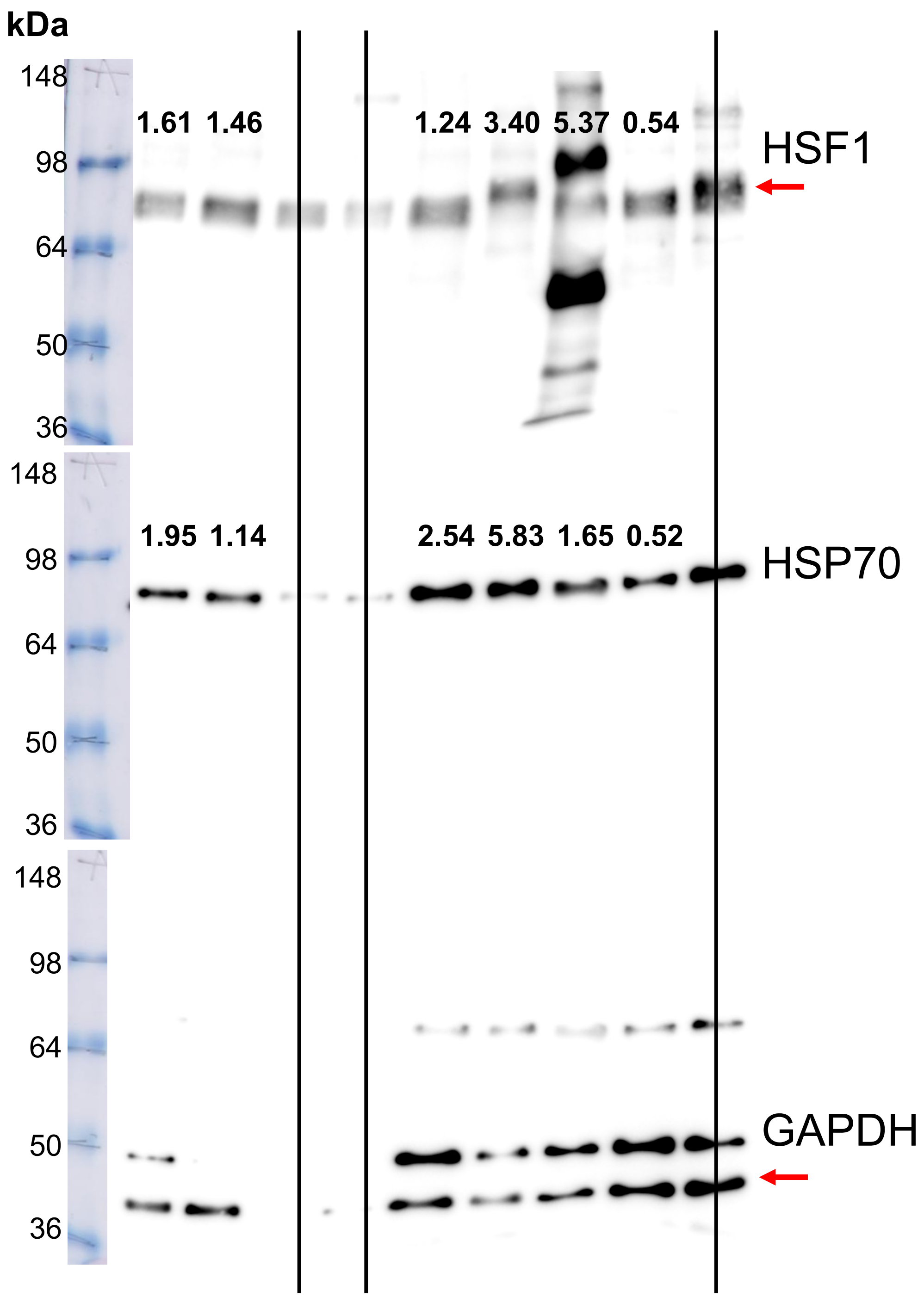

Deleted lanes represent a loading error and were not included in the manuscript.

Figure 4D

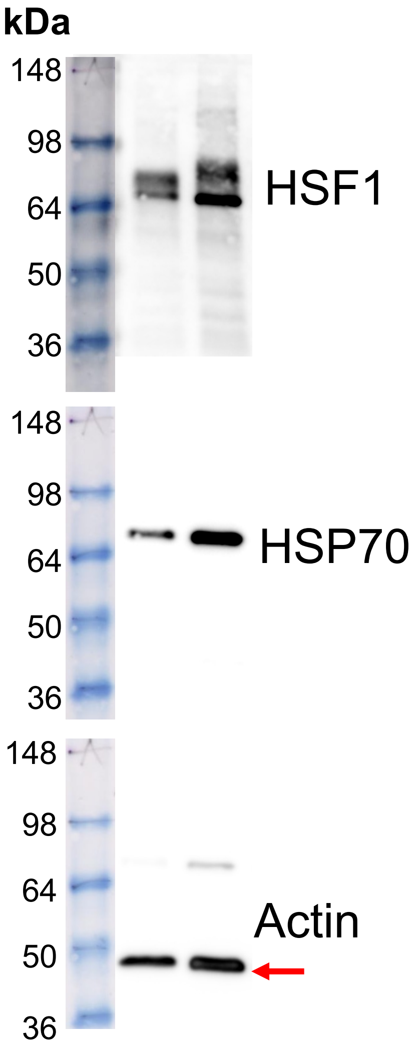
kDa
148
98
64
50
36
HSF1
148
98
64
50
36
HSP70
148
98
64
50
36
Actin

Figure 5B

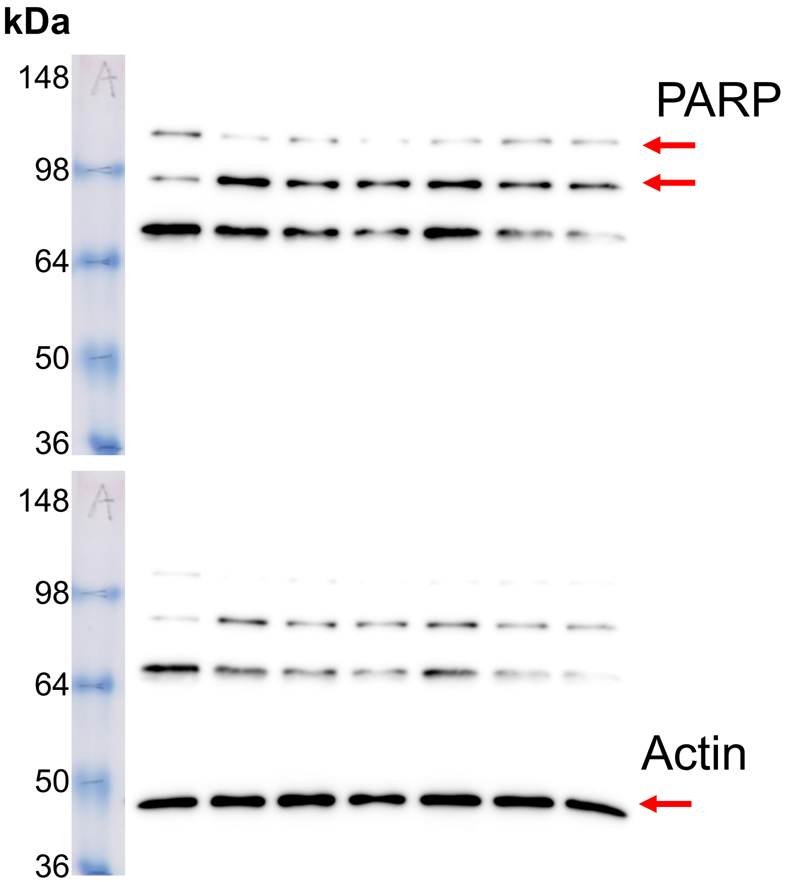

Figure 5C

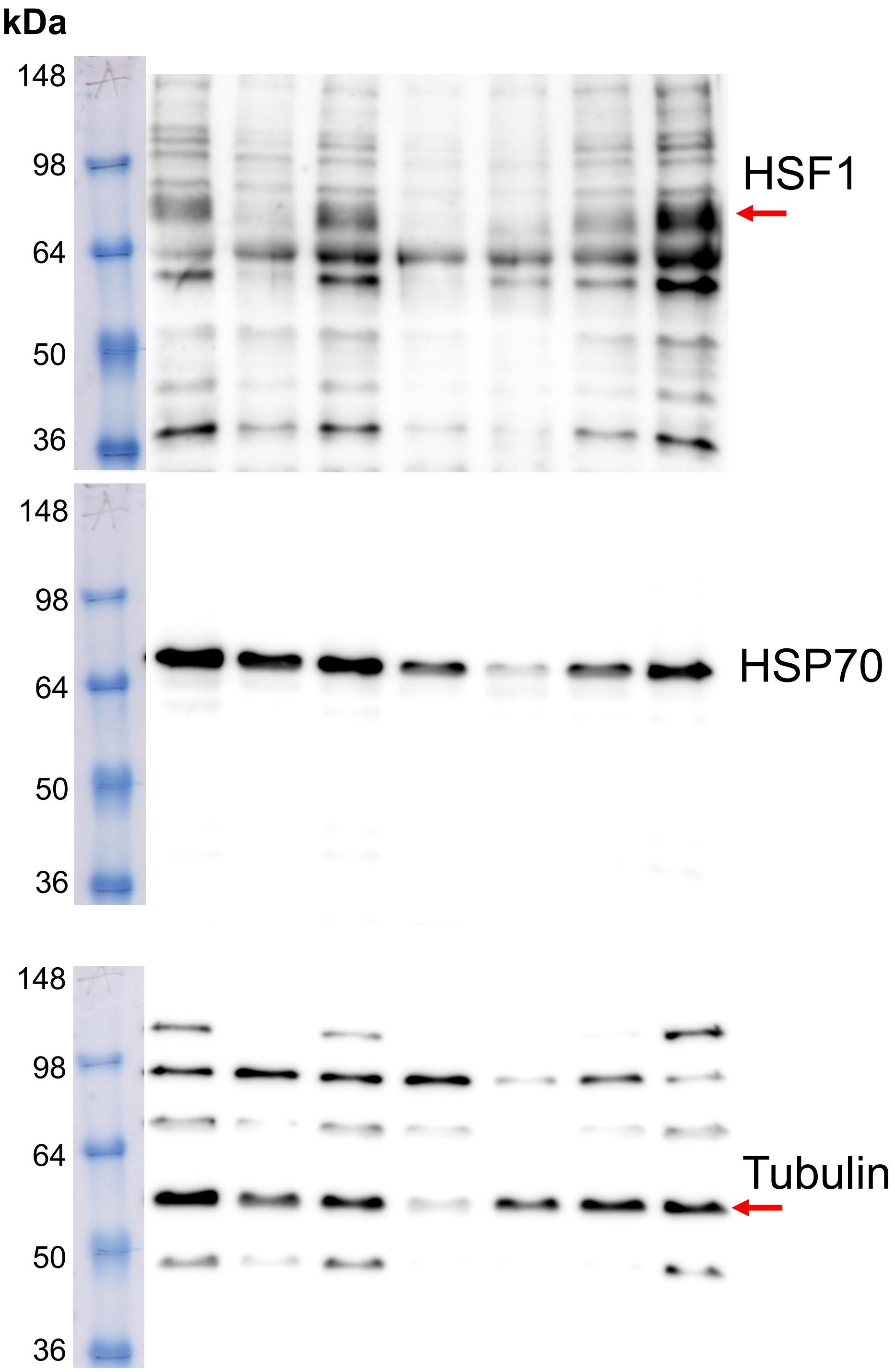
kDa
148
98
64
50
36
HSF1
148
98
64
50
36
HSP70
148
98
64
50
36
Tubulin
